# Automated Detection of Central Retinal Artery Occlusion Using OCT Imaging via Explainable Deep Learning

**DOI:** 10.1016/j.xops.2024.100630

**Published:** 2024-10-16

**Authors:** Ansgar Beuse, Daniel Alexander Wenzel, Martin Stephan Spitzer, Karl Ulrich Bartz-Schmidt, Maximilian Schultheiss, Sven Poli, Carsten Grohmann

**Affiliations:** 1 Department of Ophthalmology, University Medical Center Hamburg-Eppendorf, Hamburg, Germany; 2 University Eye Hospital, Centre for Ophthalmology, University Hospital Tübingen, Tübingen, Germany; 3 Department of Neurology and Stroke, University Hospital Tübingen, Tübingen, Germany; 4 Hertie Institute for Clinical Brain Research, University Hospital Tübingen, Tübingen, Germany

**Keywords:** AI OCT, CRAO, Deep learning retina, OCT imaging, Ophthalmology deep learning

## Abstract

**Objective:**

To demonstrate the capability of a deep learning model to detect central retinal artery occlusion (CRAO), a retinal pathology with significant clinical urgency, using OCT data.

**Design:**

Retrospective, external validation study analyzing OCT and clinical baseline data of 2 institutions via deep learning classification analysis.

**Subjects:**

Patients presenting to the University Medical Center Tübingen and the University Medical Center Hamburg-Eppendorf in Germany.

**Methods:**

OCT data of patients suffering from CRAO, differential diagnosis with (sub) acute visual loss (central retinal vein occlusion, diabetic macular edema, nonarteritic ischemic optic neuropathy), and from controls were expertly graded and distinguished into 3 groups. Our methodological approach involved a nested multiclass five fold cross-validation classification scheme.

**Main Outcome Measures:**

Area under the curve (AUC).

**Results:**

The optimal performance of our algorithm was observed using 30 epochs, complemented by an early stopping mechanism to prevent overfitting. Our model followed a multiclass approach, distinguishing among the 3 different classes: control, CRAO, and differential diagnoses. The evaluation was conducted by the “one vs. all” area under the receiver operating characteristics curve (AUC) method. The results demonstrated AUC of 0.96 (95% confidence interval [CI], ± 0.01); 0.99 (95% CI, ± 0.00); and 0.90 (95% CI, ± 0.03) for each class, respectively.

**Conclusions:**

Our machine learning algorithm (MLA) exhibited a high AUC, as well as sensitivity and specificity in detecting CRAO and the differential classes, respectively. These findings underscore the potential for deploying MLAs in the identification of less common etiologies within an acute emergency clinical setting.

**Financial Disclosure(s):**

Proprietary or commercial disclosure may be found in the Footnotes and Disclosures at the end of this article.

Acute central retinal artery occlusion (CRAO) results in painless, acute, and severe monocular vision loss, with an estimated incidence of ca 2 to 6 per 100.000 person-years.^1^^,^^2^ Despite its rarity, patients in the emergency department demand rapid professional assessment and accurate diagnosis to implement the most effective treatment strategies. Reperfusion therapy, effectively utilized in neurology for the treatment of acute cerebral ischemic strokes, can also be considered for the treatment of CRAO.^3^^,^^4^ The correct execution of reperfusion therapy in cerebral ischemic stroke relies heavily on neuroimaging analysis of morphological changes via computed tomography or magnetic resonance imaging.^3^^,^^4^ Recent advancements in machine learning algorithms (MLAs) have introduced promising new tools for enhancing the assessment and treatment evaluation of ischemic strokes in the brain.^5^

The efficacy of intravenous thrombolysis within the first 4.5 hours after the onset of CRAO is currently being investigated in multiple international prospective randomized trials, such as the REVISION, TenCRAOS, or THEIA trial.^6^, ^7^, ^8^ Given its ischemic pathophysiology, CRAO is closely linked to vascular risk factors, and its occurrence is often associated with increased comorbidities, such as acute stroke or myocardial infarction.^9^ As of today, there is no established method for automatically detecting CRAO or accurately determining its onset, which could significantly enhance diagnosis, and treatment evaluation, and improve overall prognosis.

Fundoscopy often serves as the initial diagnostic tool to identify characteristic retinal changes of CRAO, such as the cherry-red spot or retinal edema.^10^ Advancements in ophthalmological imaging technology, such as high-resolution and normalized OCT devices, have expanded the potential for improved detection and monitoring of retinal conditions. There is compelling evidence that through OCT imaging one can objectively and manually identify local pathophysiological morphological changes within the critical timeframe for thrombolysis. An early biomarker is the increased hyperreflectivity of the inner retinal layer; additional studies have also highlighted the relevance of gradual structural changes, such as ischemia, edema, and subsequent atrophy of the retina.^11^, ^12^, ^13^, ^14^, ^15^, ^16^, ^17^, ^18^, ^19^

The application of MLAs to the analysis of ophthalmological entities with emergency clinical character has been widely stated, as with fundoscopy regarding retinal vein occlusion or papilledema.^20^^,^^21^ Building on this research, we investigated the application of MLAs to detect CRAO through OCT imaging. This approach aims to automatically preprocess and distinguish specific local pathophysiological morphological changes in the retina, marking a novel application of imaging technology for this purpose.

## Methods

### Data

We utilized a retrospective anonymized data set obtained from 2 university medical centers in Germany, specifically the University Medical Center Tübingen and the University Medical Center Hamburg-Eppendorf, for training, validation, and testing. The data set was split into 3 classification categories: healthy control, CRAO, and differential diagnoses of subacute vision loss. The ground truth of the OCT images was carefully labeled by a retina expert.

The OCT images included a diverse patient group, including both women and men, with ages ranging from 21 to 98 years from rural and urban communities. Three-dimensional volume OCT scans for analysis and the respective 2D slides for analysis, were collected using a Spectral-Domain OCT (Spectralis OCT, Heidelberg Engineering) device at the time of the patients’ emergency visit to the departments.

For inclusion criteria, we defined as participants being >21 years old with no prior ocular surgery. Additionally, we only integrated images of participants with sufficient high-quality OCT imaging, as a Spectralis Q-value (decibels) >12. The type of scan for the CRAO group and most other scans is defined to be a horizontal dense volume scan with usually a size of 512 columns, 496 rows, and 19 frames. The fixation point was the macular and if no fixation was possible an external fixation light was used with the fellow eye. The average slice thickness was 0.226 mm. All OCT scans were normalized according to the criteria in Table 1 (hyperparameters, 300 pixels [px] × 300 px). All images have been extensively screened, graded, and preclassified by a retina specialist. No other significant OCT findings were described. Patients with a quality control threshold beneath a Q-value of 12 (decibels), significant secondary OCT findings, aged ≤21 years, or prior ocular surgery were excluded.

**Table 1:** Hyperparameters Used for Training and Validation

Hyperparameter	Variable
Image size	300 px × 300 px
Layers	1, 16, 1
Criterion	CrossEntropyLoss
Optimizer	Adam
Learning rate	5 × 10^–5^
Batch size	4
Early stopping epoch	5

px = pixel.

Of the 50 CRAO patients, 44 had no other retinal conditions. Two patients had early-stage dry age-related macular degeneration (small drusen), which was not relevant for OCT measurements. One patient had a nonsignificant epiretinal membrane at stage 1, and another patient had mild diabetic retinopathy without diabetic macular edema. A more detailed list of the CRAO cohort is detailed in Table 2.

**Table 2:** Central Retinal Artery Occlusion Cohort Characteristics Summary

Diagnosis	
Arterial hypertension	68%
Atrial fibrillation	2%
Type 2 diabetes mellitus	16%
Cancer	10%
Chronic kidney disease	2%
Prior stroke	12%
Prior myocardial infarction or coronary heart disease	4%
Obesity	4%
Valvular heart disease	24%
Smoker	14%
Oral contraceptive	2%
Carotid artery stenosis or dissection	4%
Risk factor	
No prior known risk factor	12%
1 prior known risk factor	30%
2 prior known risk factor	40%
≥3 prior known risk factors	18%

Our algorithm was targeted to analyze a 3-classification task: CRAO (n = 50), a control group consisting of healthy control (n = 79) combined with the contralateral eye of the CRAO participants (n = 50) if graded without significant findings and differential emergency diagnoses with (sub) acute monocular vision loss. For the latter category, we selected central retinal vein occlusion (n = 9), diabetic macular edema (n = 8) and nonarteritic ischemic optic neuropathy (n = 9). The average estimated time between the onset of CRAO via patient medical history and clinic assessment was 13.2 hours. Twelve examples were examined within 4.5 hours after symptom onset.

### Model

We employed Python (ver. 3.9.7 Python Software Foundation) for data infrastructure and all statistical analyses. For developing our deep learning framework PyTorch on a Quadro RTX 8000 GPU (NVIDIA) was utilized for this project.^22^

We applied transfer learning by choosing a pretrained deep learning residual network (ResNet) convolutional neural network model with a total of 18 layers.^23^ The structure of the model begins with an initial convolution layer, then employs max pooling with a 3 × 3 kernel. After this, there are 4 blocks of convolutional layers, each including 2 residual units and 2 convolutional layers per residual unit. After the convolutional blocks, the network employs a global average pooling layer, for spatial dimension reduction to 1 × 1 size with a single fully connected layer that outputs the results for our classification at the end.

ResNet-18, known for its “shortcut connections,” addresses performance issues related to vanishing gradients and divergence by supporting the integration and analysis of image data.

We used adaptive moment estimation (Adam) with a learning rate of 5 × 10^–5^ to update and optimize the network weights. The data were utilized by minibatches of 4 throughout the training, validation, and test dataloader. For our multiclass classification, the algorithm produced 3 outputs. The model, pretrained on the ImageNet data set, includes >1000 classes with millions of images, and utilized CrossEntropyLoss as the criterion.^24^ The loss demonstrated quantitatively the distance between prediction and true value. During training, the loss functions that operate on logits give a numerically more stable result than probabilities. The criterion applies the softmax function to the logits to obtain log loss. An early stopping method was applied for results without improvement after 5 epochs, minimizing overfitting within a maximum of 30 epochs. To conclude, the outputs of our model are logits as the final operation is a linear transformation without any additional activation functions. For training and validation, gradients were calculated and model weights updated. The evaluation modus was activated after each training epoch for validation, although the gradient update was excluded. After this tuning process of the model based on its performance at the validation set, the best set of weights was chosen, and prediction capabilities were evaluated on the holdout or test set. Test results were aggregated and used for later evaluation. A summary of hyperparameters chosen for the model tuning is presented in Table 1.

### Preprocessing

For the DICOM volume data set, the fovea centralis was identified by selecting the relative middle slice from its 3D-OCT volume scan. The filtered image was saved in PNG format and loaded into the MLA using our custom PyTorch dataloader. The selected image slides were cohesively augmented by normalization, resizing (384 px × 384 px), center cropping (300 px × 300 px), and being shifted and rotated before being processed by the model eventually. We utilized the open PyTorch albumentations module for transformation preprocessing.^25^

### Nested Cross-Validation

The entire data set of 293 images has been split, while being stratified for balancing the 3 classes, randomly into 5 relatively evenly distributed folds, hence a fivefold cross-validation. Splitting the data into their respective folds allowed us to later precisely evaluate the performance to generalize to an independent data set. To further support a more accurate and unbiased estimation of the model performance, we deployed a nested approach, with an inner train and validation loop and an outer test loop. For improving the generalization capabilities of the model, regarding the relatively limited quantity, iterated through every fold through a nested fivefold cross-validation approach, every fold has been used in the training, validation, and test set respectively exactly once.

We further distributed the participants’ anonymous IDs, and none were given in 2 different folds, preventing cross-fold data leakage, as some entities allowed the usage of both eyes. The data set was then distributed into training, validation, and test data sets respectively, with an approximate 80%/20% distribution and loaded in via the respective dataloader.

### Evaluation

For our 3-class multiclassification task, we chose the area under the curve (AUC) as the eventual evaluation metric. This metric approach is especially suitable for binary classifications and can be utilized for multiclass classifications via one-vs-all/one-vs-rest approaches. The AUC calculates the performance of 1 class, for example, CRAO cases versus the other 2 classes combined. Area under the curve measures the capability of the model to effectively discriminate between our classes. An outcome of 1.0 represents a complete discrimination detection and 0.5 represents the worst performance, equal to a random prediction of classes.

During the training and validation phases, we optimized the model’s weights, which were then saved for the subsequent testing phase. The optimal weights for testing were selected based on the average “one-vs-all” AUC value across all classes, with each class contributing equally.

During the subsequent test phase, the model’s calculated predictions will demonstrate its ability to correctly classify previously unseen data. For each batch from the test dataloader, the images were forward-passed through the model to gain the raw output logits for each class. Through the application of a softmax function, given output logits are converted into normalized values between 0 and 1, which can be interpreted as probabilities. Probabilities are easier to interpret and are crucial for decision-making, particularly in setting prediction thresholds and evaluating overall classification performance.

Regarding our nested cross-validation strategy, the results of the inner (training/validation) phases were used solely to fine-tune the weights. In the outer loop, with the respective holdout test data set, results were aggregated separately from each fold and averaged across multiple folds to better estimate generalization. This averaged AUC provides a summary metric of the entire mode, throughout all cross-validation iterations.

To compute the AUC 95% confidence interval (CI) for both sensitivity and specificity, we incorporated all classes of this multiclass data set. This cross-validation approach iterated over each of the 3 classes for each fold, and the mean was computed across folds to represent overall performance robustness. Upper and lower bounds of the 95% CI were calculated to depict uncertainty.

For each sample, sensitivity and specificity were calculated. In detail, the receiver operating characteristic curve was used to derive the false positive rate, true positive rate, and matched thresholds. The optimal threshold for sensitivity (true positive rate) and specificity (1 – [false positive rate]) were determined by identifying the point on the receiver operating characteristic curve closest to the top-left corner (0;1).

The mean was calculated as the overall pooled estimate. For the 95% CI, no direct percentile was utilized; instead, the mean and standard deviation (SD) of the true positive rate across the different folds were used. This approach provides true sensitivity and specificity values within the given intervals with 95% probability, helping to assess uncertainty around these estimates. This method is particularly advantageous when dealing with limited sample size or when variability in estimates needs to be addressed.

Furthermore, we calculated the average values for sensitivity, specificity, and AUC across multiple classes. For each chosen metric and class, the mean, as well as the lower and upper bounds of the 95% CI, was calculated to allow for a more thorough evaluation of potential uncertainties.

### Feature Map

Feature maps were integrated to explore the location of features, especially imprinting for the decision-making of the model. For this, we implemented the GRAD-CAM module in PyTorch.^26^ For result depiction, we randomly selected 1 picture multiple test sets, with correct prediction after argmax implementation to previously returned values.

## Results

### Data Characteristics

This retrospective study cohort consisted of 155 individuals and a total of 293 mobilized scans.

In the CRAO group, 54% were male and 46% were female. For the differential diagnosis group, 47% were male and 52% were female. Both groups had a minimum age of 43 years. The maximum age was 93 years for the CRAO group and 98 years for the differential diagnosis group. The mean age for the CRAO group was 73 years (SD, 10 years), whereas it was 71 years (SD, 15 years) for the differential diagnosis group (34 scans). The control group, which did not exhibit significant findings, consisted of 50 contralateral eyes OCT scans from CRAO patients, as well 159 OCT scans from an additional control cohort. This additional physiological subcohort had a male-to-female ratio of 55% to 45%, with a mean age of 47 years (SD, 22 years; range, 21–89 years).

### Model Performance

**Figure 1:**
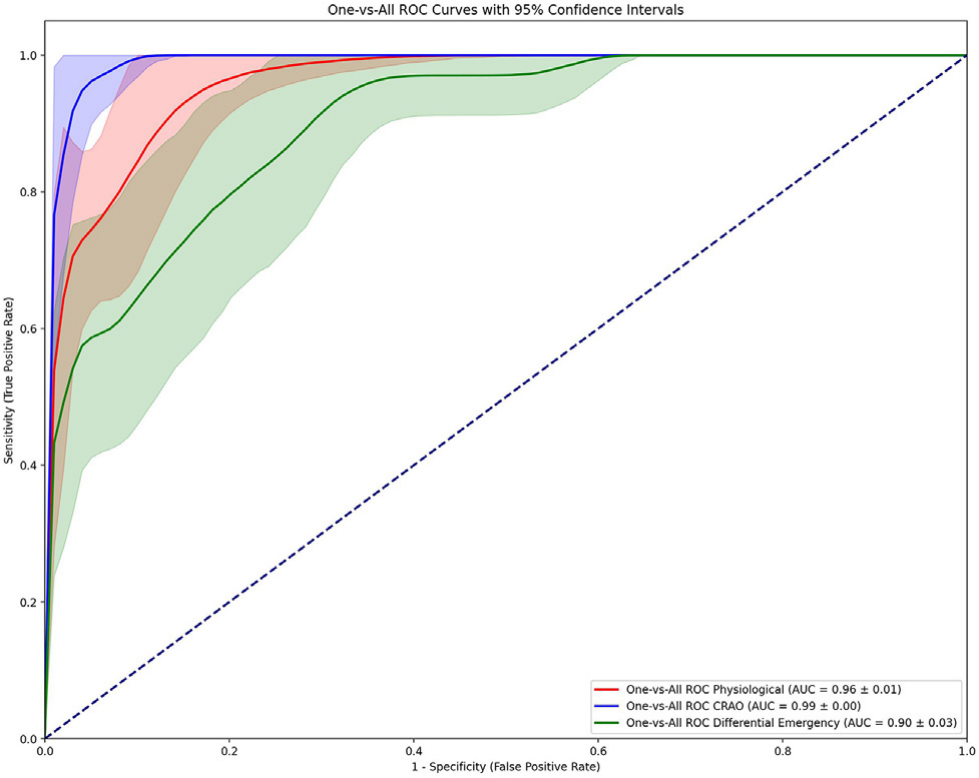
Receiver operating characteristic (ROC) curves demonstrating the capabilities of our deep learning model to distinguish the 3 different classes using 2-dimensional OCT images. An “One-vs-All” approach is employed, where each of the given classes is tested against the other 2 classes respectively. AUC = area under the curve; CRAO = central retinal artery occlusion.

In our nested 5-K Fold Cross-Validation Method analysis, we illustrate the model’s performance through 3 separate graphs in Figure 1. Each graph represents the AUC curve for 1 class versus the other 2 in a one-vs-all calculation, as AUC calculations display binary classification tasks. The return and depiction of such a figure allow for the comparison of the overall prediction capabilities of the model.

In our evaluation, all 3 classes demonstrated very high AUC values, all above 0.90. The CRAO class exhibited the best predictions with an AUC of 0.99 (95% CI, ± 0.00), followed by the control group with an AUC of 0.96 (95% CI, ± 0.01) The class for differential diagnoses with the fewest correct predictions still showed a very high AUC of 0.90 (95% CI, ± 0.03). The model demonstrated robust prediction capabilities via 2D-OCT slides, especially for CRAO, but for the other 2 classes.

Further exploration of the 95% CI curves allows for the mean calculation sensitivity, specificity, and AUC for each class. The control class achieved high results across all metrics with a sensitivity of 0.89, specificity of 0.89, and AUC of 0.96. The CRAO class received the highest values across all 3 metrics with a sensitivity of 0.96, specificity of 0.97, and AUC of 0.99. The differential diagnosis class, although having the lowest values among the 3, still showed strong results with a sensitivity of 0.79, specificity of 0.83, and AUC of 0.90.

### Attention Map

**Figure 2:**
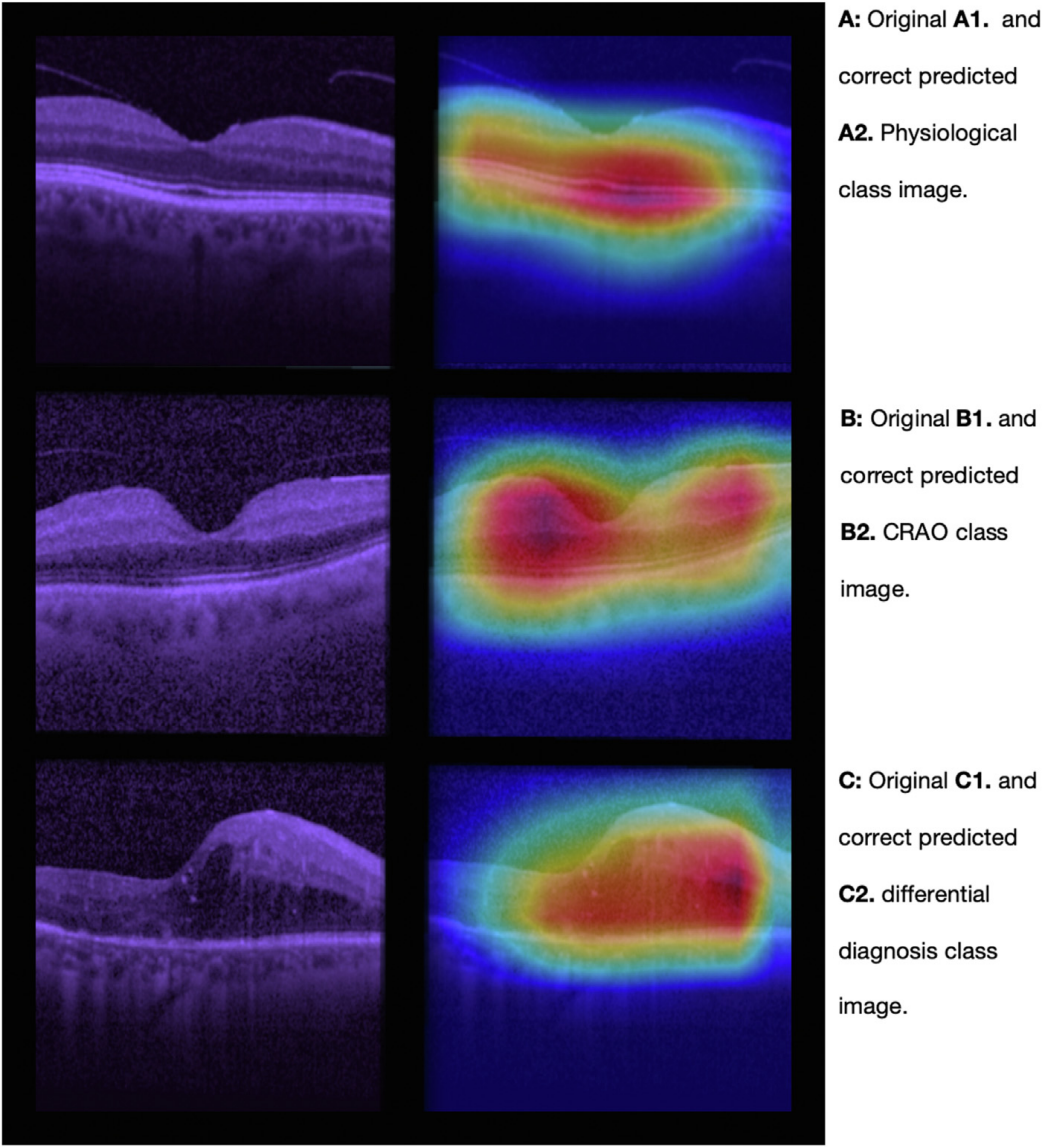
A selection of 2 dimensional OCT images, which serve as inputs for our deep learning model (shown on the left) and the GRAD-CAM feature map calculations (displayed on the right). One sample picture of each of the 3 classes has been randomly selected. The 3 figure subparts represent (**A**) physiological class, depicting normal retinal strucuture; (**B**) CRAO class, showing distinctive occlusion-related abnormalities; and (**C)** differential class, displaying other (sub) acute retinal pathologies. CRAO = central retinal artery occlusion.

The feature map, as shown in Figure 2, was obtained using the GRAD-CAM approach. One correct predicted image was randomly selected for each class. The color intensities could be interpreted as for class physiological (Fig 2A), that the macula and its surrounding spatial area are especially emphasized for its calculations. Regarding the pathomorphological changes to class CRAO (Fig 2B) and the class differential diagnosis (Fig 2C), the model seems to focus more on changes in the middle retinal layer, particularly on the periphery of the macula. The images in Figure 2 show the original input (Fig 2A, A1) before entering the MLA on the left, and the GRAD-CAM predictions (Fig 2A, A2) on the right.

## Discussion

### Findings and Interpretation

We developed, validated, and tested an MLA to detect CRAO from OCT imaging using a data set consisting of 293 eyes including CRAO cases, their partner eye, healthy controls, and a range of differential diagnoses with (sub) acute vision loss. The effectiveness of this triple multiclass classification was assessed using a nested five fold cross-validation method to ensure broad generalizability and robustness. Our analysis demonstrated a high discriminative power, with AUC values of 0.96 for controls, indicating strong identification of healthy cases; 0.99 for CRAO, showing high detection capabilities; and 0.90 for differential diagnoses, indicating a lower yet highly significant effectiveness.

The integrated feature map analysis visualized the regions of interest the model considered important for its predictions by providing insights into the MLA's decision-making process. For correct classification of control images, the MLA primarily focused on the outer retinal membranes and choriocapillaris segments reflecting its ability to recognize normal anatomical features. In contrast, for CRAO cases, the model highlighted a broader area, including regions from the inner membrane to the choriocapillaris suggesting a complex pattern of pathomorphological changes that the algorithm identifies. The derived feature map visualization of the automatically classified pictures by our convolutional neural network algorithm supports the recently published data reporting that layer thickness, as well as the opacity, are of interest to describe pathomorphological development within the acute phase of CRAO.^11^^,^^17^

With the mobilized differential etiologies, the result underscores the potential of MLAs for automated and accurate classification in ophthalmology, enabling CRAO classification with a practicable data set in the emergency setting. Our investigation highlights the use of pretrained MLAs for detecting less common conditions like CRAO in small data sets through OCT imaging. Our promising initial results suggest that MLAs could have significant future applications in ophthalmological emergencies.

Machine learning algorithms have been a key topic in recent research for a broad spectrum of clinical computation questions. Various research has provided information supporting the possibility of MLA classification to a huge range of etiologies from diabetic retinopathy over the retinal detachment to retinal vein occlusion via fundus or ultrawidefield color fundus pictures.^27^, ^28^, ^29^ OCT images were utilized for correct classification of a spectrum of findings with pathological characters, such as cystoid macular edema, epiretinal membrane, or macular hole.^30^

Possible advantages of the construction of clinical decision support systems by MLA implementation are broad. Besides its direct and imminent diagnosis capabilities, MLAs offer low implementation and upkeeping costs for supporting clinical decision-making. This could indirectly support young ophthalmologists in decision-making regarding diagnosis and monitoring. More experienced ophthalmologists could be supported by the enhancement in intelligent prescreening assessments. Especially regions with a lower distribution of experienced ophthalmological experts could benefit through the support of more intelligent automatic local- or telemonitoring-driven systems.

### Limitations

For future research, several challenges need to be addressed. The current limitations come from the given relatively small size of the data set and the limited range of differential diagnoses available in the emergency clinic setting. Expanding the data set to include a wider variety of patients, considering factors such as age, ethnicity, and the diversity of imaging equipment, could significantly enhance the validation of the model’s performance. A larger collection of CRAO images captured within the critical window for potential thrombolysis treatment would be particularly valuable, potentially facilitated by prospective multicenter studies. A major limitation is the usage of only high-quality imaging for classification, which may limit the algorithms' application to practical clinical data. This could be addressed by incorporating additional methodologies for quality control prescreening. With the nested five fold cross-validation method, we maximized our model and data set for generalization and robustness; future studies should include data from various OCT devices. Complementary analyses could compare OCT images with other modalities such as fundus photography or combine them, optimizing MLA multiclass detection in ophthalmic tasks.

### Outlook

Exploring other imaging techniques, such as fundus photography, widefield photography, 3D-OCT volumes, and OCT-angiography, could provide comprehensive insights into CRAO, its treatment, and the estimation of the possibility of treatment with intravenous thrombolysis within an acceptable time frame in an emergency setting. Integrating MLA with a multimodal approach could lead to more accurate diagnoses and better patient outcomes. Fundus photography with artificial intelligence features could serve as a cost-effective screening tool for CRAO, especially in emergency departments lacking OCT imaging capabilities or hospitals that are not linked to an ophthalmology department. As health care delivery and accessibility face challenges, such as the declining availability of medical professionals, particularly in rural regions, the integration of MLAs could strengthen interdisciplinary communication and improve patient assessments.^31^^,^^32^

### Summary

In summary, our study marks the exploration of using an MLA to analyze a mixed data set of imaging from a spectrum of acute clinical cases, important differential diagnoses, and a control group for the detection of CRAO. We demonstrated the MLA’s promising capabilities in accurately identifying CRAO, significantly aiding in diagnosis and triaging for potential thrombolysis therapy in emergency settings, where rapid and accurate decision-making is critical.
